# One Less Lead Link?: Exposure–Hypertension Association Not Replicated in Young Children

**Published:** 2006-04

**Authors:** Cynthia Washam

For two decades, scientists have known that lead exposure can induce hypertension in lab animals. More recent studies suggest it might also promote hypertension in adults. But little was known about the metal’s effects on blood pressure in children. Now researchers who studied 780 lead-exposed children for five years report seeing no indication that lead raises blood pressure in young children **[*EHP* 114:579–583; Chen et al.]**.

Lead’s most widely documented effects are neurological. Exposure diminishes intelligence and alters behavior. Young children are particularly vulnerable to these effects because their nervous systems are still developing. Children are exposed primarily through paint particles in household dust and outdoor soil contaminated with lead from paint and industrial and motor vehicle emissions. Lead exposure in the United States plummeted after the 1978 ban on lead paint, when the CDC reported that 88% of children aged 1 through 5 had blood lead levels above the level of concern of 10 micrograms per deciliter (μg/dL). By 2000, that rate had dropped to 2.2%.

The researchers originally set out to determine whether treatment with the oral chelating agent succimer would improve lead-exposed children’s scores on behavioral and cognitive tests. They recruited 780 children at clinics in Baltimore, Cincinnati, Philadelphia, and Newark. All were between 12 and 33 months of age and had moderately high blood lead levels of 20–44 μg/dL. Succimer was given to 396 children in the randomized, double-blind study. The remaining 384 children were given a placebo.

Succimer lowered blood lead levels dramatically, but there was no change in test scores. So the researchers opted to examine the data for blood pressure changes.

Clinicians had measured the children’s blood pressure every time they tested blood lead—immediately before the study and 7, 28, and 42 days after the start of each of three 26-day rounds of treatment. Measurements also were taken every three to four months for five years following treatment.

The only difference noted was a 1-mmHg increase in systolic blood pressure between one and five years after treatment—but only in the succimer group. The researchers considered this change insignificant. Diastolic pressure remained unchanged for both the succimer and placebo groups.

The team acknowledges that lead exposure might still cause hypertension years or even decades after exposure. This, combined with lead’s known neurological effects, renders the metal an important contributor to the global burden of disease.

